# Design and Analysis of a Continuous and Non-Invasive Multi-Wavelength Optical Sensor for Measurement of Dermal Water Content †

**DOI:** 10.3390/s21062162

**Published:** 2021-03-19

**Authors:** Mohammad Mamouei, Subhasri Chatterjee, Meysam Razban, Meha Qassem, Panayiotis A. Kyriacou

**Affiliations:** 1 Deep Medicine, Oxford Martin School, University of Oxford, Oxford OX1 2JD, UK; 2 Research Centre for Biomedical Engineering, City University of London, London EC1V 0HB, UK; Subhasri.Chatterjee.2@city.ac.uk (S.C.); meysam.razban.1@city.ac.uk (M.R.); Meha.Qassem@city.ac.uk (M.Q.); P.Kyriacou@city.ac.uk (P.A.K.)

**Keywords:** skin hydration, optical sensor, near infrared spectroscopy, Monte Carlo simulation

## Abstract

Dermal water content is an important biophysical parameter in preserving skin integrity and preventing skin damage. Traditional electrical-based and open-chamber evaporimeters have several well-known limitations. In particular, such devices are costly, sizeable, and only provide arbitrary outputs. They also do not permit continuous and non-invasive monitoring of dermal water content, which can be beneficial for various consumer, clinical, and cosmetic purposes. We report here on the design and development of a digital multi-wavelength optical sensor that performs continuous and non-invasive measurement of dermal water content. In silico investigation on porcine skin was carried out using the Monte Carlo modeling strategy to evaluate the feasibility and characterize the sensor. Subsequently, an in vitro experiment was carried out to evaluate the performance of the sensor and benchmark its accuracy against a high-end, broad band spectrophotometer. Reference measurements were made against gravimetric analysis. The results demonstrate that the developed sensor can deliver accurate, continuous, and non-invasive measurement of skin hydration through measurement of dermal water content. Remarkably, the novel design of the sensor exceeded the performance of the high-end spectrophotometer due to the important denoising effects of temporal averaging. The authors believe, in addition to wellbeing and skin health monitoring, the designed sensor can particularly facilitate disease management in patients presenting diabetes mellitus, hypothyroidism, malnutrition, and atopic dermatitis.

## 1. Introduction

Dermal water content is an important biophysical parameter that is interrelated with epidermal barrier function [1,2] and the desquamation process [1,3]. At the molecular level, water structure and bond formation also influence skin attributes such as dermal elasticity [4,5] and skin aging where water binding has been found to change into a tetrahedron form over time [6,7]. The skin’s outer layer, known as the stratum corneum (SC), acts as a primary barrier to the external environment, and is responsible for regulating the rate of water evaporation through skin and its water-retaining capabilities [3,8].

Measurements of dermal water content are often essential in both dermatological and cosmetic investigations, and can be carried out using a variety of non-invasive techniques. Most common are electrical capacitance measurements of skin moisture [9,10], and assessment of skin barrier function through closed-chamber instruments designed to measure transepidermal water loss (TEWL). However, both techniques are susceptible to environmental influences [9], have an indirect relationship with the parameter of interest (and hence provide arbitrary outputs), and can lack sensitivity, which as a result, can produce erroneous readings [11,12,13]. In addition, such devices usually require several minutes of equilibration time in order to stabilize. Alternative techniques such as Raman and attenuated total reflectance (ATR) spectroscopy, and confocal Raman microscopy (CRM) have also been employed for the analysis of skin hydration and permit non-invasive assessment of multiple skin parameters. CRM, in particular, is characterized by high measurement accuracy and the ability to obtain detailed depth profiles of water distribution within the SC layer and hydrogen bonding state of water molecules [14,15,16]. However, despite the advantages of such techniques, these are often large, expensive, commercially unavailable, and their outputs can be difficult to interpret.

In recent years, we have employed near-infrared (NIR) spectroscopy in comprehensive analysis of skin hydration in the optical window of 750–2500 nm, with results demonstrating a clear capability and accuracy of the technique for estimation of dermal water content. Efforts are now focused on the design and development of a transcutaneous sensor based on NIR technology that is specific to measurement of skin hydration. NIR was the preferred choice of method because absorption bands of water, proteins, and lipids inside the skin are visible in NIR spectra, and can be used to deduce information pertaining to various aspects of skin health such as hydration [17,18,19,20,21,22,23,24,25], and can differentiate between bound and free types of water inside the skin [18]. In turn, this allows a more precise method of measurement, and an added advantage is that NIR spectroscopic instrumentation can easily be equipped with fiber optic probes for in vivo measurements at various anatomical sites. Although there has been interest in improving this technique for the purpose of skin hydration measurements [26,27], a reliable handheld or portable measuring instrument of this type does not yet exist.

**Figure 1:**
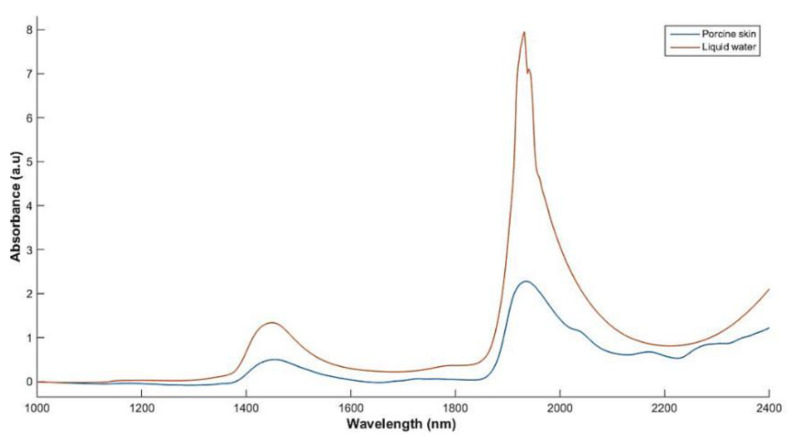
A typical NIR spectrum of liquid water versus that of porcine skin.

From the literature [18] and our previous work [28], it is known that a typical NIR spectrum of skin, such as that shown in Figure 1, is dominated by two bands around 1450 and 1900 nm, attributed to the combination of OH and HOH vibrations of water, and additional bands around 1200 and 970 nm that also relate to dermal water. This property can be exploited to build a multi-wavelength optical sensor that emits light at the wavelengths of interest, and thereby eliminate the necessity of using benchtop instruments with broad lamp sources and the generation of wide range spectra. NIR spectra of porcine skin also include absorption bands relating to CH and NH functional groups from lipid and protein constituents of skin. These include overtones of alkyl CH groups in skin lipids at around 1760 and 1730 nm, and a combination band from the same group around 2300 nm [18,28]. Bands attributed to NH bonding are typically observed at 2050 and 1500 nm, with a further C=O combination band at 2180 nm, as well as a shoulder-like character around 2000 nm representative of bound water [18,20,28].

Given the characteristics highlighted above, this paper reports on the development and evaluation of a digital multi-wavelength optical sensor that performs continuous and non-invasive measurement of dermal water content. The sensor was designed to detect optical signals at four wavelengths in the NIR region that relate to skin hydration, and was evaluated against both gravimetric and spectrophotometric measurements in a desorption experiment, with multivariate techniques being applied for data analysis and interpretation.

In order to evaluate and standardize an optical sensor, it is of utmost importance to understand the underlying light–tissue interactions pertaining to the sensor geometry (i.e., wavelengths, shape and size of the optical source and detector, source–detector separation, etc.) and the tissue region of interest (ROI). The sensor performance relies on the wavelength-dependent scattering and absorption properties of the ROI, which varies with tissue water content. Thus, for the purpose of providing comprehensive analysis, a robust model of light–tissue interaction was developed and evaluated using the Monte Carlo method. Monte Carlo is a well-known method used to simulate light–tissue interactions and produces accurate and reliable outcomes [29]. Though several Monte Carlo investigations have been carried out with different human skin ROI, no published literature is found on Monte Carlo simulation with porcine skin in relation to skin hydration. This in silico analysis, along with the aforementioned in vitro evaluation, provides a useful tool in the design and optimization of novel optical skin hydration sensing technology.

## 2. Materials and Methods

### 2.1. Monte Carlo Modeling of Optical Interactions with Porcine Skin

**Figure 2:**
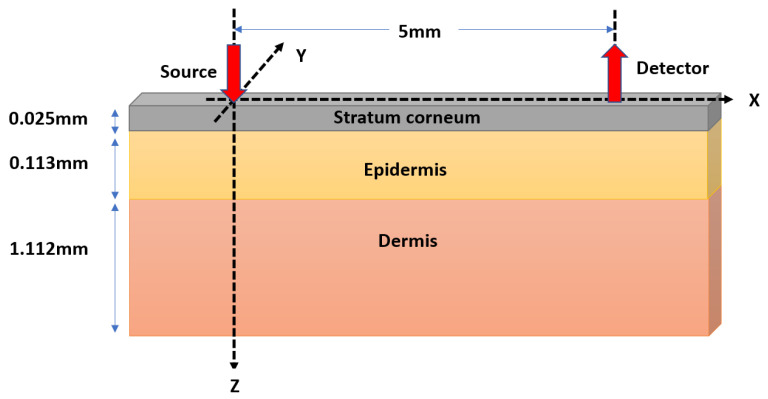
Schematic of the Monte Carlo model. The semi-infinite tissue volume with the finite thickness of 1.25 mm consisting of three layers are presented in the 3D Cartesian coordinate geometry. The optical source (red downward arrow) is simulated at the origin of the coordinate system, and the detector (red upward arrow) is simulated at a distance of 5 mm from the source.

A Monte Carlo model of optical interaction with porcine skin was developed and executed at 970, 1200, and 1450 nm. Typically, the thickness of porcine stratum corneum varies between 20–26 μm and that of epidermis varies between 30–140 μm, with dermis, the thickest skin sublayer, varying between 0.6–3 mm [30]. Considering the dermal to epidermal thickness ratio to be 10:1 [30], and the total thickness of 1.25 mm, the thickness of the three sublayers were calculated as—stratum corneum (0.025 mm), epidermis (0.113 mm), and dermis (1.112 mm). The simulated tissue volume had a semi-infinite width. The stratification of the simulated tissue volume is illustrated in Figure 2.

The scattering in epidermis and dermis are governed by keratin and collagen fibers, respectively. The scattering coefficients of keratin and collagen are similar [31]. The scattering coefficient of skin at 970, 1200, and 1450 nm are 28.64, 26.72, and 24.83 mm^−1^, respectively [31,32]. While preparing the samples, blood was removed from the tissue so that the only absorber present in the tissue was water. The absorption properties of the stratum corneum and epidermis were calculated using the formula of baseline tissue absorption coefficient [29]. Originally, this equation was developed for human skin, however, in the absence of blood, the baseline tissue properties, which depend on the structural architecture of porcine and human skin, are similar [30].

In the model, the volumetric distribution of the baseline tissue and water absorption coefficient were considered for calculating the dermis absorption coefficient [29]. The volume fraction in the final measured (i.e., dehydrated) tissue sample was considered to be 0, and then the hydrated tissue volume was simulated by adding up the desorbed water contents in the reverse order. Table 1 illustrates the water correlation of the measured weight and the volume fraction used in Monte Carlo model, and the details of the dermal absorption coefficients at the sensor wavelengths. The absorption properties of the tissue layers, on the other hand, vary with wavelengths and the water concentration. The effective absorption coefficients were calculated utilizing the volumetric absorbance distribution of the absorbers present in the tissue layers. The blood volume distribution was considered homogeneous in the dermis, and the dermal water contents were varied in 12 steps as mentioned before. The effective absorption coefficient of the dermis ($\mu_{a_{dermis}}$) was calculated utilizing the following equation:

(1) $$\mu_{a_{dermis}}\left(\lambda \right)=V_{w_{dermis}}\times \mu_{a_{water}}\;\left(\lambda \right)+\left(1-V_{w_{dermis}}\right)\times \mu_{a_{baseline}}\left(\lambda \right)$$

where $V_{w_{dermis}}$ represents the volume fraction of water at the three consecutive layers; $\mu_{a_{water}},\;\mu_{a_{baseline}}$ represent the wavelength-dependent absorption coefficients of water and baseline tissue. A baseline absorption at 5% and 20%, respectively, was discussed in our previous publication [29]. With this consideration, at 970, 1200, and 1450 nm, the stratum corneum absorption coefficients were 0.0149, 0.0074, and 0.004 mm^−1^; and the epidermal absorption coefficients were 0.0801, 0.1623, and 4.2934 mm^−1^, respectively.

In a reflectance sensor geometry, an optical source illuminating a Gaussian beam of 0.5 mm radius and a circular detector with 0.4 mm radius were simulated; the source and detector being separated by a distance of 5 mm. Detailed steps of the Monte Carlo simulation and the geometry of the model can be found in our previous publication [29]. A large number of photon packets (~10^9^–10^10^) were computed in order to obtain a high level of accuracy. A 64-bit operating system with an installed memory of 24 GB and an Intel Xeon CPU (2.40 GHz, two processors, Intel, Santa Clara, CA, USA) was dedicated for the simulation. The MATLAB (Mathworks, Inc., Natick, MA, USA) platform was chosen for coding and a multi-thread programming environment was used for facilitating the simulation.

### 2.2. Design and Development of a Hydration Sensor

**Figure 3:**
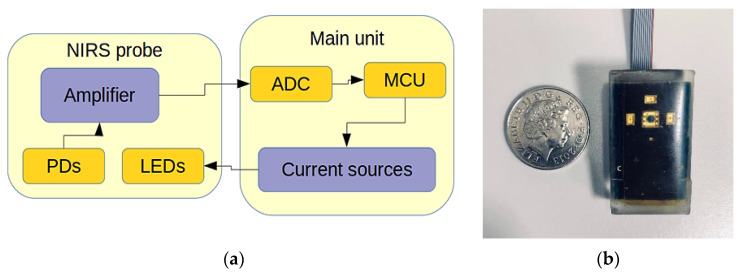
(**a**) Hydration sensor system structure, and (**b**) the hydration sensor probe.

The general system structure of the multi-wavelength sensor is illustrated by the block diagram in Figure 3. The system is comprised of two separate modules; the probe which contains the light sources, detection, and amplification, and the main module that incorporates analogue to digital conversion, current sources, and USB connection for data transfer. A detailed description of the sensor can be found in [33].

### 2.3. In Vitro Experiment

The study was approved by the Senate Research Ethics committee at City, University of London prior to experiments. Experimental setup was designed to simultaneously record the optical absorbance using the Lambda 1050 Spectrophotometer (PerkinElmer Corp, Waltham, MA, USA) and the developed hydration sensor. The reference values for water content were recorded using a TR-24 electronic precision balance (Denver Instrument GmbH, Göttingen, Germany).

A sample of porcine skin was acquired from an abattoir immediately after slaughtering. The sample was then cut using a scalpel into a 4 cm × 6 cm rectangular shapes of roughly 1–1.5 mm thickness, and then placed inside an environmental chamber (Model: KMF 115, Binder GmbH, Tuttlingen, Germany) at 96% relative humidity (RH) and 25 °C for 48 h. This ensured that maximum hydration levels were reached.

In order to mimic the natural dehydration process through the outermost later of the stratum corneum, customized sample containers were designed and 3D-printed with a single exposure side that isolated the internal sample and ensured that the desorption process occurred only through the outermost layer of the skin.

**Figure 4:**
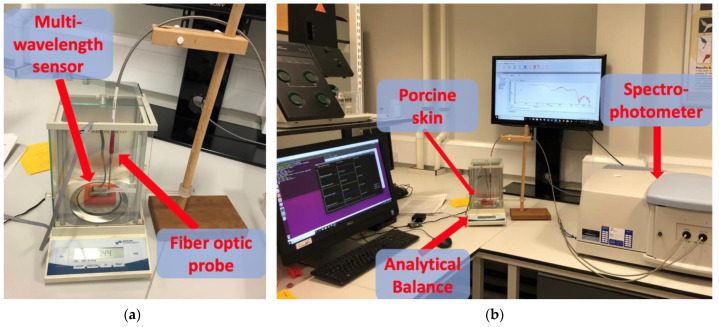
In vitro experiment setup. (**a**) The placement of the fiber optic probe and the designed sensor on the sample. (**b**) The connection of the probes to lambda 1050 and PCs.

Once fully hydrated, the skin sample was removed from the chamber and fitted inside the customized container before being placed on the analytical balance. The weight of the container was recorded prior to the insertion of the skin sample, and this value was later deducted from weight recordings and used to calculate values of dermal water content. The probe of the hydration sensor and the fiber optic probe connected to the Lambda 1050 spectrophotometer (PerkinElmer, Waltham, MA, USA) were both placed on the exposed surface of the skin sample and clamped in place for the remainder of the experiment (Figure 4).

Once the set up was complete, weight and optical measurements were initiated, and measurements were made over a 3-h period as the sample underwent desorption.

At twenty-four hours from the start of the experiment and when the specimen was found to be fully dry, a final weight measurement was recorded, reading 1.1183 g. The water content was then approximated by deducting this dry weight value from all weight measurements.

### 2.4. Instrument Settings and Data Analysis

During the 3-h desorption process, data collection was performed in the following way:

1. Intermittent weight measurements were recorded every 20 min, enabling the calculation of dermal water content.
2. DC optical reflectance readings were recorded using the novel hydration sensor at every 30 s throughout the 3-h desorption process.
3. A total of 40 reflectance spectra in the range of 872–2100 nm were collected in 3 h (every 4.5 min). Spectral acquisition was performed using the following instrument settings; InGaAs detection between 872 and 1800 nm, and PbS between 1800 and 2100 nm. The gain and response time for the InGaAs and PbS detectors were set to 3/0.2 s and 1/0.2 s, respectively. The attenuator settings were set to 1% for the reference beam and 100% for the sample compartment. This was done in order to reduce noise for high absorption values. Furthermore, an initial baseline correction of 100% transmittance and 0% absorption was also added.

The efficacy and performance of the developed sensor in measuring dermal water content was analyzed through direct comparison of optical output against the Lambda 1050 spectrophotometer. In this case, gravimetric measurements, obtained from weight measurements using an analytical balance, served as the ground truth of water content, and reflectance spectra acquired with the high-end spectrophotometer were used to benchmark optical measurements. Hence, the analysis evaluated the extent to which the same level of accuracy can be obtained using a portable multi-wavelength optical sensor, and consisted of the following steps:

1. Benchmarking optical measurements of water content with full-range spectra: The full-range optical spectra obtained via Lambda 1050 were preprocessed and subsequently fit to the interpolated weight measurements as a proxy to skin water content.
2. Investigations into the consistency of output readings from the novel skin hydration sensor and the benchtop spectrophotometer.
3. Development of a multivariate model to predict dermal water content using the skin hydration sensor, the results of which are compared to the Lambda 1050.

## 3. Results

### 3.1. Monte Carlo Characterization of the Developed Sensor

**Figure 5:**
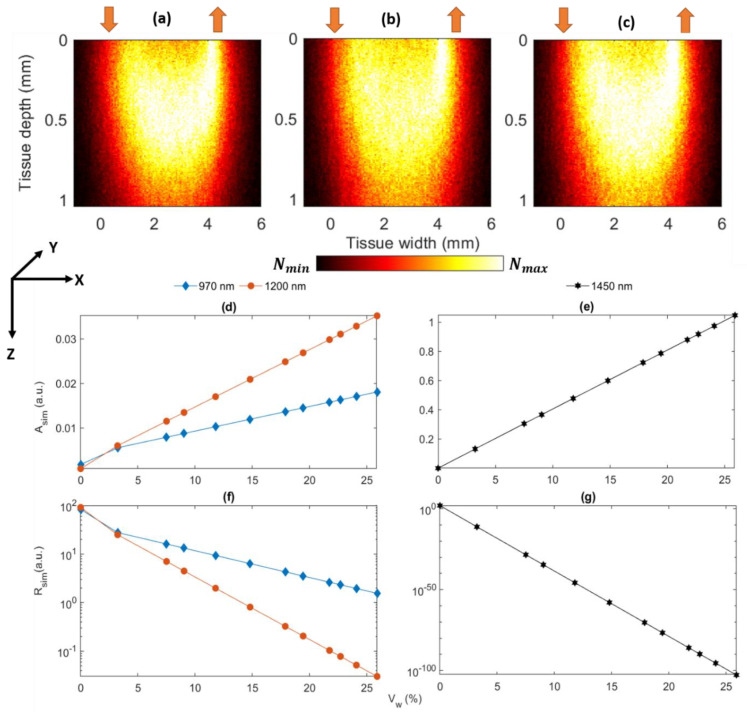
Monte Carlo simulation results at the wavelengths 970, 1200, and 1450 nm. The light–tissue interaction profiles are shown in (**a**–**c**). In the reflectance sensor geometry, the source (upward red arrow) and the detector (the downward red arrow) are separated by a distance of 5 mm. The simulations were carried out with the maximum hydrated skin at all wavelengths. Tissue depth and width are presented along the *z*- and *x*-axis, respectively. The variations in the simulated absorbance *Asim* and reflectance *Rsim* (presented in the logarithmic scale) with the increasing dermal water volume *Vw* (expressed in percentage) are shown at 970 and 1200 nm in (**d**,**f**), and at 1450 nm at (**e**,**g**), respectively.

Simulated light–tissue interaction profiles at 970, 1200, and 1450 nm are presented in Figure 5. As shown in Figure 5a–c, no significant change is visible in the interaction profile as the scattering coefficient values do not vary significantly within the three wavelengths. Optical pathlength, which depends on the scattering coefficient of the tissue, does not vary with the water concentration. The mean depth (i.e., the characteristic depth) penetrated by photons at all three wavelengths are <1 mm.

Shown in Figure 5d–g are the absorbance (*Asim*) and reflectance (*Rsim*) which were simulated at the three wavelengths for the 12 different blood volumes (volume fraction X 100%). The results for 1450 nm are shown separately to avoid improper scaling issues. As apparent from these figures, tissue absorbance increases with an increase in water volume. This is true for all simulated wavelengths and indicated by a significant positive slope at 1450 nm which exhibits a strong water absorbance band. Both the linear increase in absorbance and the exponential decay in intensity (shown as linear plots in the logarithmic scale) resulting from an increase in water concentration are shown to obey the rules of the Beer–Lambert law.

Moreover, changes in absorbance resulting from variations in dermal water concentration were simulated. The baseline stratum corneum and epidermis water concentration were considered based on our previous work [29]. Recent studies have demonstrated the concentration variation of water in stratum corneum towards dermis [14]; incorporation of such detailed parameters may immensely improve the current model. Nevertheless, both simulated and experimental data relating to absorbance and reflectance are highly comparable and demonstrate the prevalence of dermal absorbance in porcine skin hydration measurements.

### 3.2. Benchmarking Optical Measurements of Weight with Full-Range Spectra

**Figure 6:**
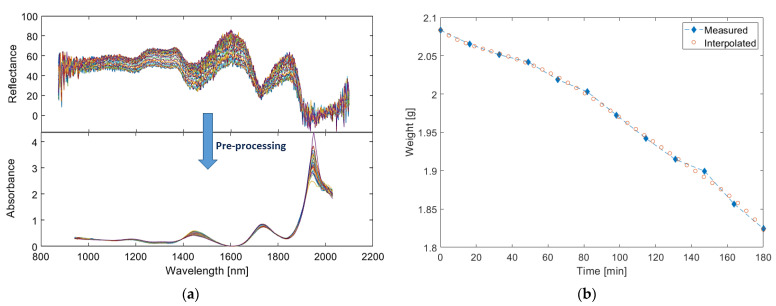
(**a**) The preprocessed spectra. (**b**) Interpolation of weights from the initial measurements.

In the first step of benchmarking optical measurements of weight with full-range spectra, the acquired diffuse reflectance data were denoised using a Savitzky–Golay filter with the polynomial order of two and the window length of 71. To account for multiplicative scattering correction (MSC), extended MSC (EMSC) was also performed with quadratic correction factor. Subsequently, the reflectance spectra are min–max normalized and transformed to absorbance spectra with log transformation (Figure 6a).

(2) $$A\propto \;log\left(\frac{1}{R}\right)$$

The resulting absorption spectra (Figure 6a) shows similar bands expected in a typical spectrum of porcine skin such as that presented earlier in Figure 1, dominated by overtone and combination bands of water at 1450 and 1920 nm, as well as the blood combination and overtone bands of CH bonding that occur between 1730 and 1760 nm, the latter of which appears to be resolved into a single band in the region of 1730–1760 nm. Spectral changes related to changes in water content are also evident as quantitative changes in absorption at the relevant bands and as peak shifts in the 1730–1760 nm region. The latter can be attributed to nonlinear changes at the 1730 and 1760 nm bands of CH bonding.

Prior to multivariate modeling, it was necessary to harmonize the time of weight readings and spectral acquisition. To address this, a polynomial of order of five was fit to the weight readings; the weight value pertaining to each spectrum was interpolated from this curve (Figure 6b). Subsequently, a partial least squares (PLS) model with five components was used to regress the interpolated weight values against the optical spectra. The number of components were selected based on the elbow point of the predict residual error sum of squares (PRESS) plot and the performance of the model was evaluated with leave-one-out cross-validation. This led to an RMSECV of 0.0149 g in the estimation of water content and a coefficient of determination of 0.9590, showing highly accurate estimates.

The results show a decrease in both weight and spectral absorption over the desorption period, where the decrease in absorption is more distinguishable at the relevant bands of 1450, 1940 nm, and between 1730 and 1760 nm. Thus, both weight and absorption decreased as the dermal water content lessened, and wavelength shifts occurred in the region of 1730–1760 nm, moving from 1735 to 1740 nm in a manner concurrent with the lessening of water content.

### 3.3. The Assessment of Agreement between the Developed Sensor and Lambda 1050 Spectrophotometer

The optical reflectance values obtained from each LED were first converted to absorbance using the same method that was applied on diffuse reflectance spectra. Due to a mismatch between the time of spectral data acquisition and the more frequent readings of the sensor, a polynomial of order five was fitted to the absorbance readings of the developed sensor for each wavelength, and the absorbance values, corresponding to the time of spectral acquisition, were interpolated.

A linear regression of the form

(3) $$A_{x}^{Lambda1050}=\beta_{0}+\beta_{1}A_{940\;nm}^{MWSH}+\beta_{2}A_{x}^{MWSH},\;$$

where A, denotes absorbance, its superscript denotes the device, and the subscript denotes the wavelength. The developed multi-wavelength skin hydration sensor is abbreviated to MWSH. The absorbance pertaining to the wavelength of 940 nm for the developed sensor is included in all three regressions (i.e.,x=1450, 1200, and 970 nm) for baseline correction. Baseline correction for the optical spectra was performed in the preprocessing step as described above. Table 2 summarizes the results.

From Table 2 it can be seen that, after correcting for baseline variations, the absorbance values of the developed sensor were highly consistent with the Lambda 1050. In addition, as expected, optical measurements at the 1450 nm band were more sensitive to variations in water content, and, as a result, outputs from both the developed sensor and spectrophotometer show a significantly higher level of agreement at this wavelength.

### 3.4. Estimation of Water Content and Comparison with Lambda 1050

Furthermore, a multiple linear model was developed to map the absorbance readings of the developed sensor to water content. The performance of the model was evaluated using leave-one-out cross-validation, and the results were compared with two models developed from diffuse reflectance spectra; the first model uses the complete spectrum (described in part a), whilst the second model uses only the four identified wavelengths. The results are demonstrated in Table 3.

Comparing the performance of the four-wavelength model and the full spectrum model trained using diffuse reflectance spectra shows only a marginal benefit in the inclusion of additional wavelengths besides the four identified wavelengths. Therefore, the four identified wavelength can adequately—almost equally—capture variations in water content. The table also clearly demonstrates that the developed sensor can not only accurately predict the concentration of water, but it also outperforms the high-end spectrophotometer that was used as a benchmark for optical estimation. The is likely due to the denoising effects of sufficient time-averaging in the sensor. The skin hydration sensor delivers rapid measurements of optical reflectance with the sampling rate of 100 Hz. Subsequently, using an exponential moving average filter, the readings are averaged and a reflectance value is obtained every 30 s. In the spectrophotometer, since the average duration of each scan is about three minutes, the same level of time-averaging cannot be performed; here each spectrum is the average of only three scans. Therefore, denoising is primarily achieved through the application of the Savitzky–Golay filter, which given the minute water-induced variation of the reflectance profile, and the presence of scattering and measurement noise (Figure 5), leads to an inferior predictive performance.

## 4. Discussion and Conclusions

An optical, multi-wavelength skin hydration sensor was designed and developed to provide a compact, continuous, inexpensive, and reliable alternative to electrical capacitance-based technologies. The design leverages three water-sensitive wavelengths (970, 1200, and 1450 nm wavelengths) and a water-insensitive wavelength (940 nm) to estimate the concentration of water in tissue.

Initial sensor evaluation was carried out using the Monte Carlo method, where an in-silico model of porcine skin was developed and explored at the selected wavelengths. The assumptions in the Monte Carlo model regarding the optical properties and thickness were based on the limited available literature. With the careful choice of the simulation parameters, the in-silico model was successfully implemented for the evaluation of the in vitro results and a comprehensive assessment of the light–tissue interactions at the three near-to-mid infrared wavelengths.

Simulation results were complemented by in vitro experiments on porcine skin to validate the design and evaluate the accuracy of its estimates. In this experiment, porcine skin underwent a desorption test, while optical measurements were acquired with the sensor and a high-end spectrophotometer. Gravimetric tests were used as a reference of porcine dermal water content. First, it was shown that the measurements of the skin hydration sensor were highly consistent with that of the high-end spectrophotometer. Secondly, we compared the performance of multivariate models developed based on the sensor and the high-end spectrophotometer. This demonstrated that the sensor can accurately predict the concentration of water and exceed the prediction accuracy of the high-end spectrophotometer. Since the spectrophotometer has a higher spectral resolution, the higher accuracy of the sensor is likely due to the denoising effect of temporal averaging; made possible by rapid reflectance measurements. Moreover, the wavelength selectivity implemented in the sensor design allows for better estimation of dermal water content as it utilizes key water absorption bands. For in vivo applications, this will permit acquisition of water-specific information with minimal influence of other biological absorbers. Future work will focus on establishing appropriate algorithms for sensor calibration in the absence of reference gravimetric data.

An accurate, low-cost, continuous, wearable skin hydration sensor can significantly improve disease management in diabetes mellitus, hypothyroidism, malnutrition, and atopic dermatitis. It can also bring about new opportunities in the wellbeing and skin health industry such as product evaluation of cosmetic formulations and skin aging. Our findings suggest that optical technologies may be the key to achieving this aim, and future efforts will focus on further assessment of the feasibility, reliability, and practical limitations of the developed sensor.

## Figures and Tables

**Table 1:** Water volume fraction in dermis (*V_w_*) and dermal absorption coefficient ($\mu_{a_{dermis}}$) used in the Monte Carlo model corresponding to the measured weight of the tissue.

Weight (g)	$V_{w_{dermis}}$	$\mu_{a_{dermis}}\;\left(mm^{-1}\right)$		
		970 nm	1200 nm	1450 nm
1.8244	0	0.0149	0.0074	0.0040
1.8567	0.0323	0.0289	0.0408	0.9276
1.8994	0.075	0.0475	0.0849	2.1487
1.9147	0.0903	0.0541	0.1006	2.5862
1.9422	0.1178	0.0661	0.1291	3.3726
1.9724	0.148	0.0793	0.1602	4.2362
2.0031	0.1787	0.0926	0.1920	5.114
2.0187	0.1943	0.0994	0.2081	5.5602
2.0417	0.2173	0.1094	0.2318	6.2179
2.0513	0.2269	0.1136	0.2417	6.4924
2.0652	0.2408	0.1196	0.2560	6.8899
2.0833	0.2589	0.1275	0.2747	7.4075

**Table 2:** Statistical analysis of the agreement between the absorbance readings from the developed sensor and lambda 1050. Each absorbance reading from Lambda 1050 is separately regressed on the corresponding absorbance values from the developed sensor. The absorbance values for the wavelengths of 940 nm is included in all regression models for baseline correction.

Wavelength (nm)	$\mathrm{Regression}\;\mathrm{Coefficient}\;{\beta_{2}}^{1}$	$\mathrm{Adjusted}\;\mathrm{Coefficient}\;\mathrm{of}\;\mathrm{Determination}\;(R^{2})$
1450	1.30 (0.078) *	0.993
1200	1.15 (0.313) **	0.91
970	0.13 (0.004) ***	0.837

^1^ The standard error is included in brackets. The statistically significant estimates with *p*-values of less than 0.01, 0.001, and 0.0001 are identified with *, **, and ***, respectively.

**Table 3:** The comparison of the predictive performance of the developed skin hydration sensor with models trained on Lambda 1050 spectra. The first Lambda 1050 model uses the whole spectrum; the second Lambda 1050 model is a multiple linear regression trained on the absorbance readings for wavelengths 940, 970, 1200, and 1450 nm. $R_{CV}^{2}$ denotes the coefficient of determination for the predicted values in the leave-one-out cross-validation.

Model	RMSECV (g)	$\mathrm{Coefficient}\;\mathrm{of}\;\mathrm{Determination}\;(R_{CV}^{2})$
Skin Hydration Sensor	0.0038	0.9975
Lambda 1050 full spectrum (#LVs = 5)	0.0149	0.9590
Lambda 1050 4 wavelengths	0.0183	0.9429

## Data Availability

Not applicable.
